# The impact of rheumatoid foot on disability in Colombian patients with rheumatoid arthritis

**DOI:** 10.1186/1471-2474-10-67

**Published:** 2009-06-15

**Authors:** Adriana Rojas-Villarraga, Javier Bayona, Natalia Zuluaga, Santiago Mejia, Maria-Eugenia Hincapie, Juan-Manuel Anaya

**Affiliations:** 1 Center for Autoimmune Diseases Research (CREA), Corporación para Investigaciones Biológicas, Universidad del Rosario, Medellin, Colombia; 2 School of Medicine, Universidad Pontifícia Bolivariana (UPB) Clínica Universitaria Bolivariana, Medellín, Colombia

## Abstract

**Background:**

Alterations in the feet of patients with rheumatoid arthritis (RA) are a cause of disability in this population. The purpose of this research was to evaluate the impact that foot impairment has on the patients' global quality of life (QOL) based on validated scales and its relationship to disease activity.

**Methods:**

This was a cross-sectional study in which 95 patients with RA were enrolled. A complete physical examination, including a full foot assessment, was done. The Spanish versions of the Health Assessment Questionnaire (HAQ) Disability Index and of the Disease Activity Score (DAS 28) were administered. A logistic regression model was used to analyze data and obtain adjusted odds ratios (AORs).

**Results:**

Foot deformities were observed in 78 (82%) of the patients; hallux valgus (65%), medial longitudinal arch flattening (42%), claw toe (lesser toes) (39%), dorsiflexion restriction (tibiotalar) (34%), cock-up toe (lesser toes) (25%), and transverse arch flattening (25%) were the most frequent. In the logistic regression analysis (adjusted for age, gender and duration of disease), forefoot movement pain, subtalar movement pain, tibiotalar movement pain and plantarflexion restriction (tibiotalar) were strongly associated with disease activity and disability. The positive squeeze test was significantly associated with disability risk (AOR = 6,3; 95% CI, 1.28–30.96; P = 0,02); hallux valgus, and dorsiflexion restriction (tibiotalar) were associated with disease activity.

**Conclusion:**

Foot abnormalities are associated with active joint disease and disability in RA. Foot examinations provide complementary information related to the disability as an indirect measurement of quality of life and activity of disease in daily practice.

## Background

The estimated incidence of foot impairment in rheumatoid arthritis (RA) varies between 85 – 91%. RA foot impairment mainly affects the forefoot and is the first symptom of the disease in 15% of RA patients. Symptoms begin in the metatarsophalangeal joints in nearly 90% of the cases [1,2]. The chronic synovial inflammation results in a secondary capsular distension, attrition of the collateral ligaments, and plantar fascia laxity, thus leading to subluxation and dislocation of the metatarsophalangeal joints and the characteristic deformities seen in feet in a situation of advanced RA [1]: hallux valgus, hammertoe, claw toe, mallet toe, as well as plantar and dorsal hyperkeratosis [3-7].

RA also involves the ankle and hindfoot joints in 30–60% of the patients. The midfoot itself is not commonly affected. However, the first metatarsocuneiform joint is the one that is often affected and consequently causes instability of the midfoot [1]. Extra-articular compromise due to severe RA may also be observed in the foot in the form of bursitis, tendinitis, tenosynovitis, fasciitis, neuropathy, skin ulceration and rheumatoid nodules [8].

RA is a disease that results in serious disability which, in turn, lowers the quality of life (QOL), increases comorbidity, causes a high economic burden and even premature mortality [9]. There are few studies showing the impact of rheumatoid foot on the patients' QOL [10]. Rheumatoid foot involvement as well as the failure to prevent or detect their progression early enough might have a significant impact on the patients' QOL. Thus, we sought to determine the main foot alterations in RA patients and to measure the impact of foot impairment on global QOL based on validated scales and their relationship to disease activity.

## Methods

### Study design and subjects

This was a cross-sectional study of 95 RA patients attending the Clinical Immunology and Rheumatology Unit of the "Clinica Universitaria Bolivariana-Corporación para Investigaciones Biológicas" in Medellin; all of them met the required American College of Rheumatology classification criteria [11]. Patients were between 18 and 80 years of age. This study was carried out between December 2005 and March 2007 in compliance with the 1993 regulation 008430 of the Ministry of Health of the Republic of Colombia. The institutional review board of the "Corporación para Investigaciones Biológicas" approved the study design, and written informed consent was received from all patients.

### Foot examination

Feet were evaluated through a detailed clinical and functional examination by two independent and experienced examiners (a foot and ankle orthopaedic surgeon and an experienced rheumatologist). The presence or absence of specific alterations, based on internationally validated definitions, was recorded on a specific standard data collection form created and agreed upon by the examiners. Definitions are as follow: hallux valgus: a static subluxation (significant structural joint displacement without total luxation) of the first metatarsophalangeal joint characterized by lateral deviation of the big toe and medial deviation of the first metatarsal [4]; hallux rigidus: a painful condition of the metatarsophalangeal joint of the big toe characterized by restricted motion (mainly dorsiflexion) and proliferative periarticular bone formation [12]; hammertoe: a flexion deformity at the proximal interphalangeal joint in which the metatarsalphalangeal joint is dorsiflexed, and the distal interphalangeal joint is in a neutral or hyperextended position [6]; claw toe: a dorsiflexion deformity of the metatarsophalangeal joint and flexion deformities at the interphalangeal joints [13]; mallet toe: a condition where the distal phalanx is flexed at the middle phalanx (with involvement of the distal interphalangeal joint) [5]; cock-up toe: an upward displacement of the toes [3]; metatarsophalangeal luxation (both partial and total): significant structural joint displacement with or without total luxation [13]; flat foot: a condition in which the arch of the foot breaks down, thus allowing the entire sole to touch the ground [14]; transverse arch flattening: transverse arch foot declination with protrusion of the metatarsal heads [15]; dorsal or plantar hyperkeratosis: well-circumscribed, painful lesions known as callosities; plantar or dorsal skin ulcers: localized injury to the skin and/or underlying tissue over a bony prominence [16]; hindfoot varus [17] and hindfoot valgus [18]; posterior tibial disfunction: evidence of a clinically apparent flatfoot upon physical examination, showing weakness with inversion of the plantarflexed foot and an inability to raise the heel while standing on a single leg [19]. Additional definitions based on pain follow: on metatarsal compression: when lateral compression was applied to the metatarsophalangeal joints in the positive squeeze test [20,21]; during movements of the forefoot [22]; during subtalar movements [23]; in tibiotalar movements [23]. Restrictions of the following were also reported: hallux plantarflexion [4]; hallux dorsiflexion[4]; lesser toes plantarflexion [5]; lesser toes dorsiflexion [5]; eversion [24]; inversion [24]; tibiotalar plantarflexion [23]; tibiotalar dorsiflexion [23].

### Clinical Variables

Besides foot examinations, all patients underwent a detailed rheumatologic examination; data were recorded on a specifically created standard data collection form. Disease activity was defined based on Disease Activity Score 28 (DAS 28). Patients with a DAS 28 score ≥ 3.2 were considered to have an active disease [25]. Assessment of QOL was based on the Spanish version of the Health Assessment Questionnaire Disability Index (Spanish HAQ-DI) [26] administered the same day as the foot examination, in addition the patients' global assessment of disease activity, a physician's global assessment of disease activity, and a visual analog scale for pain. Laboratory tests were evaluated and each of the following was classified as positive or negative: high (≥ 28 mm/h) erythrocyte sedimentation rate (ESR), and high (≥ 0.5 mg/dL) C reactive protein (CRP) serum levels. The rheumatoid factor (RF) was measured by turbidimetry (Beckman, Brea, CA); titers >40 U/ml were considered positive [27]. Third generation anticyclic citrullinated peptide (anti-CCP3) antibodies were measured by using an ELISA kit (QUANTA-Lite, INOVA, San Diego, CA) and following the manufacturer's protocol; titers >60 U were considered positive [28].

### Statistical analysis

Statistical analyses were done using the SPSS statistical package version 15 (SPSS Institute, Chicago). Associations between foot deformities, disease activity, and disability were assessed by means of a univariate analysis using X^2 ^test or Fisher's exact tests when the factors were dichotomous; or *t*-tests were utilized for assessing mean differences when the factors were continuous (data not shown). Then, two stepwise logistic regression models adjusting for age, gender and duration of disease were applied. DAS 28 >3.2 (Defining active disease) and HAQ (II–III Class defining high disability score) were used as dependent variables in the two models. As independent variables, the models included all the specific foot alterations recorded in the foot examinations which were significantly associated with disease activity or disability in univariate analyses. Adjusted odds ratios (AOR) were expressed with their 95% confidence intervals (CI). The level of significance was defined as *p*-value < 0.05. Patients who had undergone previous foot surgery were excluded in order to lower the probability of a type II error (β) of accepting a null hypothesis from a lack of association between deformities (surgically repaired) and activity and disability.

## Results

The main demographic and clinical characteristics of the 95 patients included in this study are shown in Table 1. Foot anomalies were observed in 78 (82%) of them. Hallux valgus (65%), medial longitudinal arch flattening (42%), claw toe (lesser toes) (39%), dorsiflexion restriction (tibiotalar) (34%), cock-up toe (lesser toes) (35%), and transverse arch flattening (25%) were the most frequent. The most common foot abnormalities observed are shown in Table 2. In the logistic regression analysis adjusted for current age, gender, and duration of disease, forefoot movement pain was strongly associated with disease activity (AOR = 14.4; 95% CI, 1.6 – 133.2; P < 0,0001) and disability (AOR = 16.6; 95% CI, 4 – 69.3; P < 0,0001). Pain from subtalar movement, tibiotalar movement, and plantarflexion restriction (tibiotalar) were associated with disease activity and disability. The positive squeeze test was significantly associated with disability risk (AOR = 6.3; 95% CI, 1.3 – 31; P = 0,023). Hallux valgus and dorsiflexion restriction (tibiotalar) were associated with disease activity (Table 3).

**Table 1:** Main Demographic and clinical characteristics in 95 RA patients

Variables	Percentage
Female	83.1% (79)
RF (+)	70.1% (63/89)
Steroid therapy	72.6% (69)
Methotrexate	80% (76)
Chloroquine	63.1% (60)
DMARDS	35.7% (34)
Tumor necrosis factor alpha inhibitor	15.8% (15)

	Mean ± SD

Current Age (years)	52.5 ± 12.4
Age at RA onset (years)	44 ± 12.4
Duration of RA (years)	9 ± 7.1
Swollen joints count	4.9 ± 5.6
Patient global assessment (VAS)	4.2 ± 2.7
Physician global assessment (VAS)	4.5 ± 2.3
HAQ	0.5 ± 0.6
DAS 28	3.7 ± 1.5
ESR (mm/hour)	30.6 ± 21.1
CRP (mg/dl)	0.9 ± 1.1

RF: rheumatoid factor (IU/ml); HAQ: Health Assessment Questionnaire; DAS 28: Disease Activity Score 28; ESR: erythrocyte sedimentation rate; CRP: C reactive protein; VAS: Visual Analogue Scale; DMARDS: Disease Modifying antirheumatic drugs

**Table 2:** Main foot abnormalities in a group of 95 patients with RA

Foot Alterations (N = 78)	%	N
Hallux Valgus	65.3	(62)
Hallux Rigidus	2.1	(2)
Hammer toe (Hallux)	0	(0)
Hammer toe (Lesser toes)	6.3	(6)
Claw toe (Hallux)	0	(0)
Claw toe (Lesser toes)	38.9	(37)
Cock-up toe (Hallux)	1.1	(1)
Cock-up toe (Lesser toes)	34.7	(33)
Metatarsofalangeal (luxation)	7.3	(7)
Medial Longitudinal Arch Flattening	42.1	(40/93)
Transverse Arch Flattening	25.3	(24/93)
Plantar/Dorsal Hyperqueratosis	76.8	(73)
Plantar/Dorsal Ulcers	0	(0)
Hindfoot Valgus	21.1	(20/94)
Hindfoot Varus	17.9	(17/94)
Posterior Tibial Disfunction	2.1	(2/94)
Painful metatarsophalangeal compression positive squeeze test	46.3	(44)
Forefoot movement pain	15.8	(15)
Subtalar movement pain	22.1	(21)
Tibiotalar movement pain	24.2	(23)
Plantarflexion restriction (Hallux)	3.2	(3)
Plantarflexion restriction (Lesser toes)	3.2	(3)
Dorsiflexion restriction (Hallux)	3.2	(3)
Dorsiflexion restriction (Lesser toes)	2.1	(2)
Eversion restriction	26.3	(25)
Inversion restriction	17.9	(17)
Plantarflexion restriction (Tibiotalar)	18.9	(18)
Dorsiflexion restriction (Tibiotalar)	33.7	(32)

**Table 3:** Foot alterations associated with disability and disease activity risk.

Foot Deformities and Alterations	Disease Activity (DAS28 > 3.2)			Disability (HAQ II – III)		
	
	AOR	95%CI	P	AOR	95%CI	p
Hallux Valgus	2.7	(1–7)	0.018	NS	NS	NS
Positive Squeeze test	NS	NS	NS	6.3	(1.3 – 31)	0,023
Forefoot movement pain	14.4	(1.6 – 133.2)	0,000	16.6	(4 – 69.3)	0,000
Subtalar movement pain	7.2	(1.6 – 34)	0,012	5.5	(1.5 – 20.5)	0,011
Tibiotalar movement pain	7.9	(1.7 – 37)	0.009	4.7	(1.3 – 17.4)	0.019
Plantarflexion restriction (Tibiotalar)	14	(1.7 – 116.1)	0.014	4.5	(1.2 – 17.1)	0.025
Dorsiflexion restriction (Tibiotalar)	4.7	(1.6 – 14.7)	0.008	NS	NS	NS

AOR: Adjusted odds ratio by age, gender and duration of disease; CI: confidence interval; DAS 28: Disease Activity Score 28; HAQ: Health assessment questionnaire

## Discussion

This study describes the main types of foot impairment in Northwestern Colombian RA patients and shows their association with disease activity and disability. Our study confirms a high rate of rheumatoid foot alterations (82%) in the patients studied herein. These results are comparable to other reports previously published [1,29].

Foot pain and secondary limitations on regular daily activities are common complaints of rheumatoid patients. However, physical examination of the foot may be omitted in routine practice. This lack of examination may be due to a reliance on the common measurement of disease activity (DAS28) that omits the feet and ankle joints. Some authors have found discrepancies in the physical examination, depending on whether foot and ankle joints are included in the examination or not, which suggests that DAS28 should be used cautiously in clinical practice when disease remission is measured [30].

Despite the fact that examination of the feet has been omitted from the evaluation of disease activity in RA patients, we recommend its use. It is an important tool for predicting disability, which is also the main predictor of poor outcome in RA patients [31]. In fact, we have shown that some rheumatoid alterations, particularly the ones related to pain during foot examination, are strongly related to disease activity as well as to high HAQ scores, disclosing a strong relationship to disability and QOL.

Although some authors suggest that disease duration does not necessarily correlate with self-reported foot pain or disability [32], in this study, we searched for main predictors of high HAQ scores among those with rheumatoid foot abnormalities. At the same time we adjusted for disease duration, to avoid bias. It must be recognized that disease duration increases the correlation between damage and disability [31].

Some disease-related components can be measured by using a single RA-specific foot-health instrument and several generic ones. A number of researchers have developed a foot impact scale to assess foot status in RA [33]. Another, the Foot Function Index (FFI), was developed to measure the impact of foot pathology in terms of pain, disability and restriction [34]. For FFI and subscales, HAQ has been the most important predictor [10]. Other studies have found that moderate-to-high foot impairment and related disability can be detected early in RA patients [35]. Based on our results, this should encourage performing a basic physical examination of the feet from the onset of the disease and during all aspects of medical care in RA patients because it might be possible to predict disability based on certain foot alterations.

One of the most interesting results of the present study is the strong association between the painful compression metatarsophalangeal positive squeeze test and HAQ disability. A previous study [36] developed a clinical model for the prediction, during the first visit, of 3 forms of the outcome of arthritis: self-limiting, persistent nonerosive, and persistent erosive. Bilateral compression pain in the metatarsophalangeal joints (positive squeeze test) is one of the predictors of persistent erosive arthritis in the model. At present, we can confirm that it is a predictor of not only poor outcome but also disability. It is important to discern that the results reported on the HAQ affect the QOL significantly and independently [37] and in fact, the baseline HAQ score was the best predictive factor for QOL as assessed by the Arthritis Impact Measurement Scales 2 that measures QOL in previous studies [38].

Among the specific foot deformities studied, hallux valgus was the most prevalent one significantly associated with disease activity. Some studies based on the use of x-ray definition revealed a correlation between hallux valgus and disease severity and at the same time, other deformities [39]. X-ray classification was not used in the current study to describe hallux valgus, but it could be implemented for a more exact approach to this deformity. Even if an X-ray measurement is lacking, it is important to emphasize the usefulness of evaluating this deformity in some way in daily practice because it is associated with a high DAS28 score. It is of interest to mention that when some authors measured hallux valgus using X-rays, the results were comparable to the judgment of a panel of experienced clinicians [40].

A significant association between tibiotalar movement pain, plantarflexion restriction (tibiotalar), dorsiflexion restriction (tibiotalar) and disease activity was found. This anatomical region is not easy to examine; this association could be related to the presence of existing inflammation (i.e., pannus and synovitis) that is not always easily found during clinical examination. In fact some authors have found poor ranges for sensitivity (55–83%), and specificity (23–46%) while looking for tibiotalar synovitis by comparing clinical examination and ultrasound with magnetic resonance imaging as a gold standard method [41].

## Conclusion

This is the first study evaluating foot impairment in this particular population of patients with RA. Foot abnormalities are associated with active joint disease and disability in this population. It is important to notice that pain score, one of the components of DAS28, is the predominant clinical assessment associated with poor health status, which is measured by using disability and QOL instruments [31]. It also has the greatest impact on individual subdimensions of the HAQ as a measure of disability [42]. Foot examinations in daily practice provide complementary information related to the disability as an indirect measurement of QOL and activity of the disease and should be systematically included to complement but not replace any of the existing measuring tools. This study should guide future research in the search for an association between corrected foot deformities under conservative (i.e., orthoses) or surgical treatment and disease activity and disability in a longitudinal design, encouraging the inclusion of a higher sample size that should have a strong statistical association with rheumatoid foot impairment.

## Competing interests

The authors declare that they have no competing interests.

## Authors' contributions

ARV conceived the study, participated in the design of the study and performed the statistical analyses. All authors carried out the clinical examination. ARV and JMA wrote the manuscript with the help of JB. All authors have read and approved the final manuscript.

## Pre-publication history

The pre-publication history for this paper can be accessed here:



## References

[B1] Abdo RV, Iorio LJ (1994). Rheumatoid arthritis of the Foot and Ankle. J Am Acad Orthop Surg.

[B2] Trieb K (2005). Management of the foot in rheumatoid arthritis. J Bone Joint Surg.

[B3] Smyth CJ, Janson RW (1997). Rheumatologic View of the Rheumatoid Foot. Clinical Orthopaedics and Related Research.

[B4] Easley ME, Trnka HJ (2007). Current concepts review: hallux valgus part 1: pathomechanics, clinical assessment, and nonoperative management. Foot Ankle Int.

[B5] Coughlin MJ (1984). Mallet toes, hammer toes, claw toes, and corns. Causes and treatment of lesser-toe deformities. Postgrad Med.

[B6] Caselli MA, George DH (2003). Foot Deformities: Biomechanical and Pathomechanical changes associated with aging, Part I. Clin Podiatr Med Surg.

[B7] Woodburn J, Stableford Z, Helliwell PS (2000). Preliminary investigation of debridement of plantar callosities in rheumatoid arthritis. Rheumatology.

[B8] O' Brien TS, Hart TS, Gould JS (1997). Extraoseus Manifestations of Rheumatoid Arthritis in the Foot and Ankle. Clinical Orthopaedics Rel Res.

[B9] Anaya JM, Pineda-Tamayo R, Gómez LM, Galarza Maldonado C, Rojas-Villaraga A, Martín J (2006). Artritis Reumatoide, Bases moleculares, clínicas y terapéuticas.

[B10] Bal A, Aydog E, Aydog ST, Cakci A (2006). Foot deformities in rheumatoid arthritis and relevance of foot function index. Clin Rheumatol.

[B11] Arnett FC, Edworthy SM, Bloch DA, McShane DJ, Fries JF, Cooper NS, Healey LA, Kaplan SR, Liang MH, Luthra HS, Medsger TA, Mitchell DM, Neustadt DH, Pinals RS, Schaller JG, Sharp JT, Wilder RL, Hunder GG (1988). The American rheumatism association 1987 revised criteria for the classification of rheumatoid arthritis. Artritis Rheum.

[B12] Coughlin MJ, Shurnas PS (2003). Hallux rigidus: demographics, etiology, and radiographic assessment. Foot Ankle Int.

[B13] Mizel MS, Yodlowski ML (1995). Disorders of the Lesser Metatarsophalangeal joints. J Am Acad Orthop Surg.

[B14] Pinney SJ, Lin SS (2006). Current concept review: acquired adult flatfoot deformity. Foot Ankle Int.

[B15] Popelka S, Vavrík P, Pech J, Veigl D (2003). Deformities of the forefoot in patients with rheumatoid arthritis-results of surgical treatment. Acta Chir Orthop Traumatol Cech.

[B16] Stewart M, Bernhard J, Cropley T, Fitzpatrick T, Freedberg IM, Eisen AZ, Wolff K, Austen KF, Goldsmith LA, Katz SI (2003). The Structure of Skin Lesions and Fundamentals of Diagnosis. Fitzpatrick's Dermatology in General Medicine.

[B17] Cracchiolo A (1997). Rheumatoid arthritis. Hindfoot disease. Clin Orthop Relat Res.

[B18] Keenan MA, Peabody TD, Gronley JK, Perry J (1991). Valgus deformities of the feet and characteristics of gait in patients who have rheumatoid arthritis. J Bone Joint Surg Am.

[B19] Myerson MS, Myerson MS (2005). Correction of the flatfoot deformity in the adult. Reconstructive Foot and Ankle Surgery.

[B20] Moder KG, Hunder GG, Harris ED, Budd RC, Genovese MC, Firestein GS, Sargent JS, Sledge CB (2005). History and physical examination of the musculoskeletal system. Kelley's Textbook of Rheumatology.

[B21] Rigby AS, Wood PH (1991). The lateral metacarpophalangeal/metatarsophalangeal squeeze: an alternative assignment criterion for rheumatoid arthritis. Scand J Rheumatol.

[B22] Coughlin MJ (2000). Common causes of pain in the forefoot in adults. J Bone Joint Surg Br.

[B23] Sizer PS, Phelps V, James R, Matthijs O (2003). Diagnosis and management of the painful ankle/foot part 1: clinical anatomy and pathomechanics. Pain Pract.

[B24] Leardini A, Stagni R, O'Connor JJ (2001). Mobility of the subtalar joint in the intact ankle complex. J Biomech.

[B25] Prevoo ML, Van't Hof MA, Kuper HH, Van Leeuwen MA, Putte LB Van de, Van Riel PL (1995). Modified disease activity scores that include twenty-eight-joint counts. Development and validation in a prospective longitudinal study of patients with rheumatoid arthritis. Arthritis Rheum.

[B26] Cardiel MH, Abello-Banfi M, Ruiz-Mercado R, Alarcon-Segovia D (1993). How to measure health status in rheumatoid arthritis in non-English speaking patients: validation of a Spanish version of the Health Assessment Questionnaire Disability Index (Spanish HAQ-DI). Clin Exp Rheumatol.

[B27] Anaya JM, Correa PA, Mantilla RD, Jimenez J, Kuffner T, McNicholl JM (2001). Rheumatoid Artritis in African Colombians from Quibdo. Seminars Artritis Rheum.

[B28] Correa PA, Tobón GJ, Citera G, Cadena J, Schneeberger E, Camargo JF, Maldonado-Cocco JA, Anaya JM (2004). Anti-cyclic citrullinated peptide antibodies in rheumatoid arthritis: relation with clinical features, cytokines and HLA-DRB1. Biomedica.

[B29] Jaakkola JI, Mann RA (2004). A review of rheumatoid arthritis affecting the foot and ankle. Foot Ankle Int.

[B30] Landewé R, Heijde D van der, Linden S van der, Boers M (2006). Twenty-eight-joint counts invalidate the DAS28 remission definition owing to the omission of the lower extremity joints: a comparison with the original DAS remission. Ann Rheum Dis.

[B31] Scott DL, Smith C, Kingsley G (2003). Joint damage and disability in rheumatoid arthritis: an updated systematic review. Clin Exp Rheumatol.

[B32] Leeden M van der, Steultjens M, Dekker JH, Prins AP, Dekker J (2007). The relationship of disease duration to foot function, pain and disability in rheumatoid arthritis patients with foot complaints. Clin Exp Rheumatol.

[B33] Helliwell P, Reay N, Gilworth G, Redmond A, Slade A, Tennant A, Woodburn J (2005). Development of a foot impact scale for rheumatoid arthritis. Arthritis Rheum.

[B34] Budiman-Mak E, Conrad KJ, Roach KE (1991). The Foot Function Index: a measure of foot pain and disability. J Clin Epidemiol.

[B35] Turner DE, Helliwell PS, Emery P, Woodburn J (2006). The impact of rheumatoid arthritis on foot function in the early stages of disease: a clinical case series. BMC Musculoskelet Disord.

[B36] Visser H, le Cessie S, Vos K, Breedveld FC, Hazes JM (2002). How to diagnose rheumatoid arthritis early: a prediction model for persistent (erosive) arthritis. Arthritis Rheum.

[B37] Haroon N, Aggarwal A, Lawrence A, Agarwal V, Misra R (2007). Impact of rheumatoid arthritis on quality of life. Mod Rheumatol.

[B38] Cohen JD, Dougados M, Goupille P, Cantagrel A, Meyer O, Sibilia J, Daurès JP, Combe B (2006). Health assessment questionnaire score is the best predictor of 5-year quality of life in early rheumatoid arthritis. J Rheumatol.

[B39] Shi K, Tomita T, Hayashida K, Owaki H, Ochi T (2000). Foot deformities in rheumatoid arthritis and relevance of disease severity. J Rheumatol.

[B40] Budiman-Mak E, Roach KE, Stuck R, Spencer F, Polizos T, Conrad KJ (1994). Radiographic measurement of hallux valgus in the rheumatoid arthritic foot. J Rheumatol.

[B41] Wakefield RJ, Freeston JE, O'Connor P, Reay N, Budgen A, Hensor EM, Helliwell PS, Emery P, Woodburn J (2008). The optimal assessment of the rheumatoid arthritis hindfoot: a comparative study of clinical examination, ultrasound and high field MRI. Ann Rheum Dis.

[B42] Häkkinen A, Kautiainen H, Hannonen P, Ylinen J, Arkela-Kautiainen M, Sokka T (2005). Pain and joint mobility explain individual subdimensions of the health assessment questionnaire (HAQ) disability index in patients with rheumatoid arthritis. Ann Rheum Dis.

